# Compensatory Belief in Health Behavior Management: A Concept Analysis

**DOI:** 10.3389/fpsyg.2021.705991

**Published:** 2021-08-27

**Authors:** Kang Zhao, Xinyi Xu, Hanfei Zhu, Qin Xu

**Affiliations:** ^1^School of Nursing, Nanjing Medical University, Nanjing, China; ^2^Faculty of Health, Queensland University of Technology, Brisbane, QLD, Australia

**Keywords:** behavior management, compensatory belief, concept analysis, health behavior, health psychology

## Abstract

**Purpose:**

This study aimed to identify the exact definition of the concept of compensatory belief (CB) and to help clinicians and caregivers to distinguish patients who tend to form such beliefs.

**Methods:**

This study is a conceptual analysis based on the strategy of Walker and Avant (2014). We retrieved studies from existing literature using databases such as PubMed, CINAHL, Wiley, and Web of Science, and extracted information from the results. No date limitations were applied.

**Results:**

A total of 35 articles were sourced for data extraction. The identified attributes of CB were as follows: cognitive coping strategy, pleasure without guilt, compensatory behaviors, automatic motivated self-regulatory process, and implicit cognition. The observed antecedents were: failure to resist desire, reluctance, a conflicting motivational state, seeking appropriate balance, and self-image maintenance. The consequences of CB were lower health-related intentions, justification of unhealthy choices, relief from cognitive dissonance, continued existence of conflict feeling, and poor long-term health.

**Conclusion:**

Compensatory belief stems from motivational dissonance or confliction. Despite causing feelings of pleasure and relief, it can negatively impact long-term health. Although CB is crucial to establish healthy behaviors, it is easily ignored in medical work.

## Introduction

Since the 21st century, researchers have been studying the impact of behavior on health outcomes. Activities and lifestyle factors such as smoking, overeating, and lack of exercise lead to an increased incidence of obesity and other chronic diseases (McGinnis and Foege, 1993), bringing about challenges to medical work. Consequently, people try to adopt a healthy lifestyle, hoping to reduce the occurrence of negative outcomes (Pinel et al., 2000). However, maintaining a healthy lifestyle proves difficult. Several studies on weight management have exemplified this—according to the National Institute of Health Technology, most dieters regained the weight they initially lost within 5 years, and half of the participants quitted exercise within 6 months (NIH, 1992, 1993). Why do people have difficulty persevering in their healthy behavioral choices and what is their reason to quit?

Before the concept of compensatory belief (CB) was proposed, most health-related research studies assumed that healthy behavior choices are generated by rational assessments (Schachter, 1982; Schifter and Ajzen, 1985), and few studies took into account the motivational factors (Hart, 1993; Blanton and Gerrard, 1997). Knäuper et al. (2004) focused on the motivational factors. They put forward that cognitive dissonance occurs when the pleasure of engaging in an indulgent behavior conflicts with the potential adverse health effects, and that the resolution of this dissonance requires a process of self-regulation. There are three different situations of cognitive dissonance and their corresponding mitigation strategies. First, in the case of a high sense of self-efficacy or a low degree of temptation, people resist this desire. Second, when the desire is very strong and becomes hard to control, people adjust their risk assessment of the behavior and conform to it. Third, in the cases where the desire for behavior is medium, a contradictory belief will be activated, which Knäuper et al. termed “compensatory health belief” (CHB), also abbreviated as “compensatory belief” (CB). In this scenario, individuals consider that subsequent healthy behaviors can compensate for the adverse effects of indulging in unhealthy activities. The nature of CB can be well explained by the following example (Hart, 1993). On the one hand, in the face of a delectable cake, one may know the dessert itself contains a huge amount of saturated fats, cholesterol, and sugar, which is harmful to our well-being. On the other hand, one has the desire to enjoy the cake and imagines how delicious it must taste. These two conflicting perceptions lead to tangled and contradictory feelings that make one think: “it is fine to eat this cake since I will work out later to burn extra calories.” In other words, one believes physical exercise can compensate for the negative effects of unhealthy foods. One uses CB to solve this “guilty pleasure” dilemma and to defend unhealthy choices. Therefore, it is precisely CB, a concept brought to light by Knäuper et al., that complicates maintaining a healthy lifestyle.

The significance of CB in health behavior management has been recognized during the past two decades. Rabiau et al. (2006) introduced a model to explain the action mechanism of CB. The model was later tested in various empirical studies, including their study on medication adherence in adolescent patients with diabetes (Rabiau et al., 2009). The study reported that emphasizing the existence of CB in the education of blood glucose monitoring and correcting their wrong ideas improved the therapeutic adherence of these patients. In addition, Radtke et al. (2014) investigated smoking-related CB under the behavior change model and improved a smoking-specific CB scale. Verger et al. (2018) studied CB in the context of influenza prevention and discovered people believe that avoiding exposure or resorting to curative treatments compensates for vaccination, which is essentially a CB. Recently, under the influence of multiple health behavior change (MHBC) vision of intervention, CB has become increasingly relevant. The rationale of MHBC consists of promoting various beneficial behaviors concurrently or in an orderly manner, within a specified period of time (Prochaska and Prochaska, 2011). Control CB can maintain multiple behavioral interventions simultaneously. Research on CB will help us to realize MHBC in medical work.

Although CB is fundamental to health behavior management, its definition remains to be heterogeneous across different studies. Only one systematic review has been written on compensatory behaviors (Buckley et al., 2019). Furthermore, most previous studies focused solely on the behaviors of concern, none have summarized the commonalities of CB among different health-related behaviors, some studies even gave controversial results (Garner et al., 2014; Sim and Cheon, 2019). Moreover, some concepts or phrases exist that have similar meanings to that of CB [e.g., moral licensing (Kouchaki, 2011) or irrational health belief (IHB) (Christensen et al., 1999)], which are likely to cause confusion. The present concept analysis was aimed at addressing these issues. By analyzing the concept of CB in this study, we hoped to gain an in-depth comprehension of its components and support the development of theories. Such understanding can help us to identify the existence of CB in patients and to learn how to overcome it.

## Methods

### Concept Selection

In the field of health behavior, promoting the development of healthy behaviors or the avoidance of unhealthy ones is undoubtedly challenging. Although CB is considered to be a key variable that may influence this process (Rabiau et al., 2006), the lack of a uniform definition makes it difficult to distinguish CB from other similar concepts. Moreover, the methods used to evaluate this concept need to be improved (Kaklamanou et al., 2013). Since further conceptualization of CB is required, we selected this concept for analysis.

### Conceptual Analysis Process

In the present study, we employed a recognized method to conceptualize CB in the context of health behavior management (Walker and Avant, 2014). Table 1 shows the eight steps of this method.

**TABLE 1:** The eight stages of the concept analysis method conceived by Walker and Avant.

**Steps in order**	**Specific content**
Step 1	The selection of a concept
Step 2	The determination of the analysis purpose
Step 3	The identification of all possible uses of the concept
Step 4	The creation of the defining attributes
Step 5	The identification of a model case
Step 6	The identification of borderline, related, and contrary cases
Step 7	The identification of antecedents and consequences
Step 8	The definition of empirical referents

### Literature Search Strategy

**FIGURE 1:**
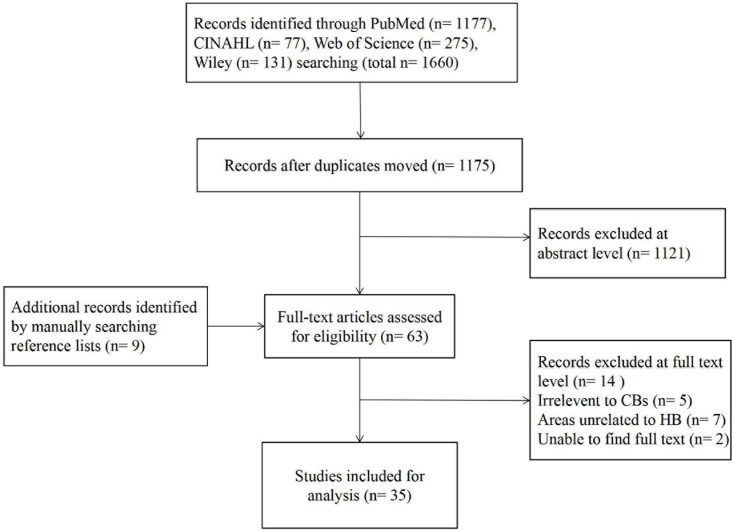
Flowchart of the study selection process of the concept analysis.

A systematic retrieval of electronic databases including PubMed, CINAHL, Wiley, and Web of Science was conducted on June 30, 2021. We used “compensatory belief” and “health behaviors” as search keywords to retrieve relevant literature and bibliography. We did not search for synonyms such as “moral licensing” and “compensatory green belief” because they are less commonly used in studies of health-related behaviors (although these are mentioned later in the related concepts section). No date limitations were applied to the search protocol. All the studies published in English focused on CB in the field of health behavior management were included. Of the original 1,660 studies that were found (Figure 1), 1,175 studies remained after excluding duplicates. Based on a title assessment, we also excluded irrelevant research, book reviews, letters to the editor, and studies published in a language other than English. Next, we thoroughly read the abstracts of the remaining studies and only included specific studies in the final analysis. The inclusion criteria consisted of at least one of the following items: definitions, contributing factors, attributes, antecedents, consequences, and/or method for CB measurement. The above information was extracted for subsequent analysis.

## Results

### Literature Included

Based on the aforementioned inclusion and exclusion criteria, 35 studies were taken into account for the final analysis, including 26 quantitative studies, 4 qualitative studies, 4 literature reviews, and 1 multiple methods study. The dates of publication range from 2004 to 2021. The studies were conducted in 12 countries, mainly distributed in Europe and North America. Study details are shown in Table 2.

**TABLE 2:** Articles selected for the final analysis.

**References**	**Country**	**Sample size**	**Research design**	**Area of focus and contribution of the study**
Knäuper et al. (2004)	Canada	634	Scale development study	Describes a psychometric scale to measure compensatory health beliefs and provides data on its reliability and validity.
Rabiau et al. (2006)	Canada	–	Literature review	Brings up a model to explain why people create compensatory health beliefs and how they employ compensatory health beliefs to regulate their health behaviors.
de Nooijer et al. (2009)	Netherlands	145	Scale testing study	Culturally adapts compensatory health beliefs scale for use in the Dutch context and assesses its psychometric properties.
Rabiau et al. (2009)	Canada	114	Scale development study.	Develops glucose testing compensatory belief scale to investigate whether compensatory beliefs regarding glucose testing predict blood glucose levels and adherence to treatment in Canadian adolescents with type 1 diabetes.
Kronick et al. (2011)	Canada	69	Longitudinal observational study	Uses experience sampling methodology to test if, for dieters who face inevitable temptations over the course of the day, compensatory thinking is predictive of caloric intake.
Radtke et al. (2011)	Switzerland	244	Scale development study	Develops a scale to measure smoking-specific compensatory health beliefs among Switzerland adolescents and to test whether compensatory health beliefs are related to a lower readiness to stop smoking.
Kaklamanou et al. (2013)	England	43	Qualitative study	Utilizes “think aloud” technique in English students to identify the kinds of difficulties that people experience when completing compensatory health belief scales and what steps will be required to develop a future reliable and valid measure of compensatory health beliefs.
Miquelon et al. (2012)	Canada	121	Prospective longitudinal study	Uses path analysis in Canadian female college students to examine how initial autonomous motivation would influence compensatory beliefs activation, goal adherence, and goal attainment over the course of a diet.
Radtke et al. (2012)	Switzerland	385	Cross-sectional model verification study	Investigates the compensatory health beliefs within the model of HAPA to examine if smoking-specific compensatory health beliefs are able to add to the prediction of the intention to quit smoking.
Ernsting et al. (2013)	Germany	851	Randomized controlled trial	Explores how German individual vaccination behavior is affected by the mediating role of compensatory belief between intention and behavior through volitional self-regulation strategy intervention.
Glock et al. (2013)	Luxembourg	107	Cross-sectional study	Investigates implicit and explicit compensatory health beliefs among German smokers and analyzes their possible influence on healthy behaviors as well as their role in predicting smoking behavior.
Poelman et al. (2013)	Netherlands	179	Scale development study	Develops the diet-related compensatory health beliefs scale (Diet-CHBs) and testing the scale’s internal consistency and validity in Dutchman.
Berli et al. (2014)	Switzerland	430	Prospective cohort study	Applies multilevel model to examine the contribution of compensatory health beliefs to the prediction of Swiss adolescents’ physical activity within the model of HAPA.
Garner et al. (2014)	America	677	Cross-sectional study	Examines whether American students’ thinness expectancies contribute significant variance in the endorsement of excessive exercise over and above binge eating, restraint, and shape and weight concerns.
Lippke (2014)	Germany	–	Literature review	Introduces a new model including compensatory belief, in order to study multiple behaviors in one model
Radtke et al. (2014)	Germany	75	Randomized controlled trail	Investigates diet-specific compensatory health beliefs in Switzerland and England dieting women within the context of the HAPA model to examine the extent to which diet-specific compensatory health beliefs contribute to dieting intentions and dietary intake.
Fleig et al. (2015)	Germany	416	Cross-sectional online study	Using the HAPA model to investigate the extent to which transfer cognitions and compensatory health beliefs contribute to single behavior theory in Europeans.
Amrein et al. (2017)	Switzerland	232	Mixed methods study	Utilizes path models to analyze the role of compensatory health beliefs within the HAPA model; applies content analysis to investigate the occurrence of compensatory health beliefs in smartphone chat groups when pursuing an eating goal to investigate the role of compensatory health beliefs for two distinct eating behaviors.
Storm et al. (2017)	Germany	790	Longitudinal randomized controlled trial	Examines the role of compensatory health beliefs in predicting fruit and vegetable consumption intentions and actual fruit and vegetable consumption among German and the Netherlands people.
Gray et al. (2018)	England	9	Qualitative study	Uses retrospective qualitative process evaluation with one-to-one semi-structured interviews to explore the mechanisms of physical activity compensation among Ireland older adults.
Matley and Davies (2018)	England	249	Cross-sectional study	Conducts an online survey in England to examine associations between alcohol-specific compensatory health beliefs and behaviors, alcohol consumption, and alcohol-specific self-efficacy.
Verger et al. (2018)	France	19	Qualitative study	Conducts semi-structured in-depth interviews of France diabetic patients to study their reasons for choosing or rejecting seasonal influenza. Some patients reported their compensatory beliefs.
Buckley et al. (2019)	Australia	–	Systematic literature review	Compiles and appraises the evidence of studies pertaining to the relationship individuals have with food and their bodies after retiring from sports.
Capstick et al. (2019)	England	6,969	Cross-sectional study	Develops and evaluates a survey-based instrument to assess people’s compensatory and catalyzing beliefs in Brazil, China, Denmark, India, Poland, South Africa, and the United Kingdom.
Dohle and Hofmann (2019)	Germany	235	Observational Study	Explores the relationship between four types of sequential health behaviors and the health status, life satisfaction, and compensatory beliefs of French people.
Martin et al. (2019)	America	198	Randomized controlled trail	Identifies the mechanisms responsible for weight compensation on American healthy overweight or obese people.
Petersen et al. (2019)	Australia	100	Non-randomized controlled trial	Tests compensatory health beliefs model by examining the influence of snack consumption (healthy, unhealthy) on type of activity selected (physical, sedentary) among Australian female undergraduate students.
Ren et al. (2019)	China	64	Cross-sectional study	Uses the adapted compensatory health belief scale to make survey, thus providing behavioral evidence for implicit beliefs and implicit behavioral tendencies toward smoking-related cues among Chinese male smokers and non-smokers.
Sim and Cheon (2019)	Singapore	23	Randomized controlled trail	Investigates the influence of the impending consumption of a meal perceived to be healthy on prior snack consumption on Singaporeans.
Amrein et al. (2020)	Switzerland	166	Prospective longitudinal study	Tests whether daily compensatory health beliefs were associated with the daily intention to quit and daily number of cigarettes smoked during smoking cessation in Swiss couples.
Forestier et al. (2020)	France	104	Cross-sectional study	Investigates whether different compensatory health beliefs predict intentions in individuals with cardiovascular diseases among French patients.
Neter and Bagants (2020)	Israel	773	Scale development cross-sectional study	Develops the compensatory health beliefs on breastfeeding scale. Attempts to neutralize or reduce the cognitive dissonance between non-nursing and optimal infant care.
Amrein et al. (2021)	Switzerland	45	Longitudinal observational Study	Applies an ecological momentary assessment design to distinguish the effects of state and trait compensatory health beliefs on unhealthy snack consumption in daily life at the between- and within-person level.
Law et al. (2021)	Australia	18	Qualitative study	Provides evidence regarding the nature of, and factors underpinning, Australian parents’ physical activity-related compensatory beliefs for their children.
Lippke et al. (2021)	Germany	–	Scoping review	Elaborates on the question as to what extent internet activity is predictive of psychological well-being based on the Compensatory Carry-Over Action Model.

### Utilization of CB in the Context of Health Behaviors

In the first study (Knäuper et al., 2004) on CB, the concept was defined as a conviction that engaging in healthy behaviors can compensate for the harmful effects of previous unhealthy behaviors. Later, scholars found that CB is related to behavioral intention, self-efficacy, and risk perception (Kronick et al., 2011; Garner et al., 2014). Then, research on CB gradually began to be combined with theoretical models such as the health action process approach (HAPA) (Fleig et al., 2015) and the compensatory carry-over action model (CCAM) (Lippke, 2014), in order to systematically explore the relationship between CB and other variables.

The abovementioned studies mainly focused on the implementation of unhealthy behaviors mediated by CB. Other studies, more oriented toward the anticipatory use of CB, have investigated people applying CB as a reason not to perform certain behaviors, such as physical exercise, glucose testing, and vaccination (Rabiau et al., 2009; Ernsting et al., 2013; Poelman et al., 2013). Besides, reports on the use of CB became more diversified, and more specific scales to measure CB were gradually produced as well. Some scholars have coined other terms to describe CB, such as “licensing” and “compensatory cognition” (Dohle and Hofmann, 2019; Lippke et al., 2021). These advances led to the discovery of other causes for CB, such as self-image maintenance and seeking appropriate balance (Dohle and Hofmann, 2019).

**FIGURE 2:**
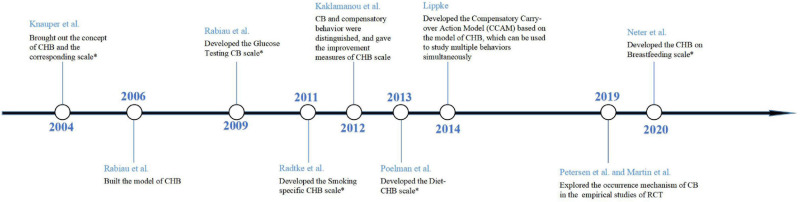
Timeline of the key changes in the development of CB. CHB, compensatory health belief; CB, compensatory belief. A “*” sign indicates that the author has developed a new scale.

In recent years, researchers have been more attentive to the nuances of this concept in different situations. Many studies have explored the relationship between CB and behavioral intention and self-efficacy by classifying patients according to the level of cognitive risk or behavioral stage (Radtke et al., 2014; Forestier et al., 2020). This allowed future interventions and management of CB to be more personalized. Recent research has also attempted to examine CB in breastfeeding and parent–child relationships (Neter and Bagants, 2020; Law et al., 2021), indicating that this concept is increasingly gaining recognition in other behavioral studies. Figure 2 shows a timeline depicting the key changes in the development of CB in the past two decades.

### Related Concepts

**FIGURE 3:**
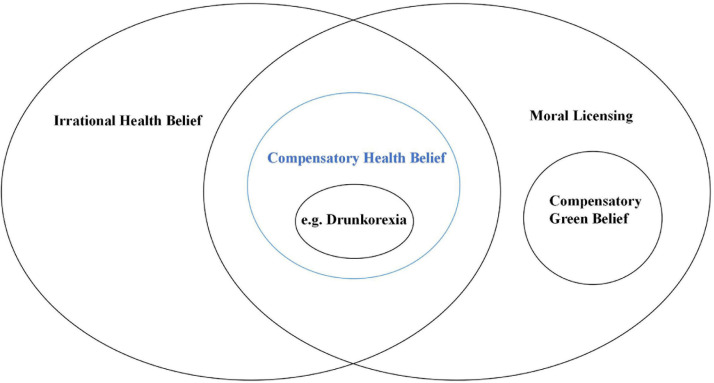
Relationship between CB and the related concepts.

Related concepts are terms close to the concept studied, but can be distinguished from the original concept by further analysis. We drew a Venn diagram to reflect the relationship between CB and the related concepts introduced in this review (Figure 3).

We uniformly called “compensatory belief” as “compensatory health belief” in this figure to differentiate them from other related concepts and to emphasize the health area of concern.

#### Food and Alcohol Disturbance

The concept “food and alcohol disturbance” (also known as drunkorexia) can be interchangeably used with the concept of CB. Food and alcohol disturbance (Choquette et al., 2018) is a type of dissonance activated when one reduces food intake for alcohol drinking to maximize drunkenness and counteract the calories of alcohol. In fact, food and alcohol disturbance is one of the dimensions in CB, and CB may also relate to other problem behaviors and psychological disorders.

#### Irrational Health Belief

Certain concepts could be easily confused with CB. Irrational health belief, which was first employed by Christensen et al. (1999), is used to reflect negative patterns of health behavior as well as health-related cognitive distortions. For example, an individual who considers the advice of their physician unnecessary may think: “this piece of advice was not useful when I had disease X; therefore, it is not useful for any other condition.” The main difference between IHB and CB is that the former just arises from outcome evaluation, while the latter is generated from conflicting motivational behaviors. In addition, IHB reflects the attributions concerning health events, whereas CB is related to self-regulation of health-related behavior.

#### Moral Licensing

Moral licensing refers to the phenomenon where individuals allow themselves to engage in morally ambiguous or immoral behavior because of past moral or socially acceptable behavior (Monin and Miller, 2001). For example, people who feel good after refuting sexist and racist comments are more likely to make discriminatory decisions. The concept of CB actually falls within the scope of moral licensing. However, moral licensing covers a wider range of fields and is mainly used to study social, economic, and managerial issues (Hofmann et al., 2014). Therefore, CB is not equivalent to moral licensing.

#### Compensatory Green Belief

Although health is the main focus of this study, CB exists in other fields as well. Compensatory green belief is a concept used in the field of environmental management (Sörqvist and Langeborg, 2019). It is frequently used to explain why people continue to damage the environment even though they are simultaneously trying to restore the environment and ecosystems. Compensatory green belief, which is also a particular type of moral licensing, can reduce the sense of guilt caused by the negative impact of detrimental behaviors on the environment.

### Defining Attributes

The key component of concept analysis is the identification of the most relevant attributes with the concept, which provides researchers with a deeper understanding. The attributes contribute to distinguishing the concept under study from similar or related concepts. The most commonly defined attributes of CB include cognitive coping strategy, pleasure without guilt, compensatory behaviors, automatic motivated self-regulatory process, and implicit cognition.

#### Cognitive Coping Strategy

Cognitive coping strategy refers to strategies that reduce cognitive dissonance by changing the attitude of an individual or adding certain reasons. When individuals are faced with temptations, there is an inconsistency between their desires and their understandings of the adverse effects of temptations, leading to cognitive dissonance. This dissonance includes fear of disease due to unhealthy behaviors, collision with self-perceptions (such as “I am a healthy person”), and discrepancy with self-expectations (such as successfully quitting smoking) (de Nooijer et al., 2009; Forestier et al., 2020). The process of solving cognitive dissonance by generating CB is called a cognitive coping strategy (Miquelon et al., 2012).

#### Pleasure Without Guilt

Individuals tend to feel guilty when they surrender to temptation. People who pursue health goals will thus be filled with negative emotions, including feeling conscience-stricken and self-denial, while enjoying the happiness triggered by unhealthy behavior (Glock et al., 2013; Matley and Davies, 2018). CB emerges at this moment as a perfect excuse for those who feel guilty to engage in pleasant behavior. This belief allows individuals to connive their unhealthy behavior without feeling uncomfortable; hence, they no longer pay attention to the negative effects of their choices.

#### Compensatory Behaviors

The emergence of CB is based on individuals believing that a “compensatory behavior” can help them maintain their health. However, people with CB do not necessarily execute compensatory behaviors. Behavior is only the medium of the belief generated by patients, so compensatory behavior is one of the attributes (Kaklamanou et al., 2013). Simultaneously, compensatory behavior should be distinguished from health behavior, since certain behaviors are indeed compensatory (Lippke, 2014; Fleig et al., 2015), but other compensatory behaviors are detrimental or cannot offset the negative effects of previous unhealthy behaviors (Amrein et al., 2017).

#### Automatic Motivated Self-Regulatory Process

Compared with those who do not feel guilty about their harmful diet or smoking habits, individuals who experience CB do aspire to be healthy—although they may not perform the compensatory behavior later, at intention level, they are attempting to self-regulate (Gray et al., 2018). According to a previous study, the level of CB improves over time after it has been formed because such belief is spontaneously generated and recognized by oneself (Fleig et al., 2015). Under the guidance of others, individuals may also compensate for temptation through subsequent compensatory behavior, but this situation is unstable (Gray et al., 2018). The belief generated under such circumstances cannot be counted as compensatory.

#### Implicit Cognition

Implicit cognition means that although the traces of past experiences cannot be self-aware, the previous experiences will potentially impact the current behavior of an individual (Wolsiefer et al., 2017). Basically, the childhood environment of an individual will exert an imperceptible influence on their ideas and cognitions. Studies have confirmed that implicit cognition leads smokers with CB to associate smoking and exercise behaviors together (Glock et al., 2013; Ren et al., 2019). Thus, implicit cognition is a bridge between unhealthy behavior and later compensatory behavior and promotes the emergence of CB.

### Cases of CB

The theory by Walker and Avant argues that cases can help illuminate and clarify concepts. Therefore, we adapted cases from specific literature into a model case, a borderline case, a related case, and a contrary case.

#### Model Case

The model case is a typical example of using a concept to demonstrate all of its defining attributes and to better illustrate the meaning of the main concept.

Max (Rabiau et al., 2009), an 18-year-old patient with diabetes, is aware that he needs to test his blood sugar before sleeping. However, he does not want to test while staying at the house of a friend during the holiday because he is ashamed of sharing with his friends that he has diabetes. Since omitting the test before bedtime is wrong, he feels nervous and anxious (**cognitive coping strategies are needed**). He has learned that some patients do not measure their blood sugar every day, but he cannot comfortably skip the test (**automatic motivated self-regulatory process**).

He thinks (**implicit cognition**) that “if I test my blood sugar twice tomorrow morning, it may compensate for the consequences of omitting it tonight.” Max cannot say where this idea originated from, but after strengthening his belief, he goes to bed with peace of mind (**pleasure without guilt**). The next morning, he tests his blood sugar twice (**compensatory behavior**) at an interval of 2 h. Ultimately, Max often replaces testing before bedtime with double measures the next morning, which will affect his diabetes and overall health.

#### Borderline Case

The borderline case is an instance that contains most (but not all) of the defining attributes of the concept.

Daniel (Gray et al., 2018), a 22-year-old undergraduate student who is losing weight, always insists on eating vegetables but seldom exercises. On a certain occasion, his roommate buys fried chicken and invites him to dine together. He wants to enjoy the meal but worries that the interruption of his diet will ruin his previous efforts (**cognitive coping strategies)**. While Daniel is hesitating, his roommate says, “Just eat it. I will go running with you to lose weight later.” Then, after Daniel happily enjoys the food (**pleasure without guilt**), they exercise for an hour that night (**compensatory behavior**). However, when Daniel passes the fried chicken restaurant again, he forces himself to eat a vegetable salad, but the dissonance of not eating fried chicken afflicts him.

In this case, Daniel does not spontaneously associate fried chicken with sports, which represents an inconformity to the attribute of implicit cognition, as well as being inconsistent with the automatic motivated self-regulatory process. Thus, Daniel did not activate CB.

#### Related Case

The related case is an example that is related to the concept but contains only part of the defining attributes of the concept.

Ms. W (Kaklamanou et al., 2013) is a 30-year-old pregnant woman. She gained a significant amount of weight during her pregnancy. One day she wants to drink high-calorie nourishing soup. Although she is prepared to face overweight after pregnancy, eating well still makes her uncomfortable. However, her mother tells her that eating better was good for her baby, so she still has the nourishing soup (**pleasure without guilt**). This case only reflects the attribute of pleasure without guilt, there is no CB in Ms. W.

#### Contrary Case

The contrary case is a representative example that is clearly different from the concept under study.

Mr. L (Gray et al., 2018) is a 50-year-old retired gymnast. He has maintained healthy eating habits for many years and never compromises on high-calorie foods. At the same time, he enjoys smoking. His friends tell him that smoking does not affect his health and he never feels guilty about smoking. In this case, Mr. L can directly refuse the temptation of high-calorie foods while simultaneously smoking without resistance or hesitation. Consequently, he does not exhibit any CB.

### Antecedents and Consequences

This section extracts the most utilized antecedents and consequences in the included references to further define the characteristics of CB. Identifying antecedents and consequences helps us to have a deeper understanding of the defining attributes.

#### Antecedents

The incidents necessary for a concept to exist or happen are called antecedents. Five antecedents of CB were identified in this analysis: (1) failure to resist desire; (2) reluctance; (3) a conflicting motivational state; (4) seeking appropriate balance; (5) self-image maintenance.

Motivation is the psychological tendency or internal drive that motivates and maintains the action of an individual and directs it to a certain goal (Capstick et al., 2019). For an individual with a health-related goal, whether he is indulging in harmful behaviors or avoiding beneficial ones, these choices will inevitably collide with their goals of pursuing health. In other words, they are in a conflicting motivational state (Rabiau et al., 2006). When they cannot avoid this conflict and struggle with behaviors, they will seek appropriate balance at the motivation level, which leads to CB. Under this circumstance, irresistible situations include the desire to eat high-calorie food, smoke, and drink alcohol (Radtke et al., 2012; Storm et al., 2017; Amrein et al., 2020). The reluctance to engage in healthy actions includes reluctance to exercise and refusing to get vaccinated (Garner et al., 2014; Petersen et al., 2019). In addition, people also attempt to maintain a positive and healthy image, for which CB becomes a suitable explanation or excuse (Dohle and Hofmann, 2019).

#### Consequences

The incidents that occur as a result of the concept are called consequences. Five consequences of CB were identified in this analysis: (1) lower health-related intentions; (2) justification of unhealthy choices; (3) relief from cognitive dissonance; (4) continued existence of conflict feeling; (5) poor long-term health.

Among these consequences, lower health-related intention and justification of unhealthy choices are frequent in people with CB (Fleig et al., 2015). The activation of CB contributes to finding suitable reasons for individuals to explain “why my unhealthy behavior is not wrong,” thus alleviating the discomfort resulting from unhealthy events and eliminating the feelings of compunction or guilt (Berli et al., 2014). Subsequently, the initial discomfort gradually diminishes, until individuals no longer need CB to alleviate their negative emotions, causing their health intention to decrease in the process (Gray et al., 2018).

The last three consequences are produced under different circumstances, according to the execution of compensatory behaviors. When compensatory behavior is displayed, the cognitive dissonance of an individual can be reduced, leading to relief (Rabiau et al., 2006). Conversely, when compensatory behavior is not carried out, the motivational dissonance of an individual will exist for a given period of time but will be eventually alleviated (Martin et al., 2019). Lastly, compensatory behavior may be implemented, but in some cases, its effects are unhealthy or do not compensate for the previous harmful choices. Therefore, the individual mistakenly thinks that the compensatory behavior eliminates all negative effects, which leads to poor long-term health (Ernsting et al., 2013).

Under the influence of CB, the behavioral patterns of individuals will develop in a negative direction, which explains the impact of CB on poor long-term health. First, CB transforms healthy behaviors that people think they need to do into choices that they consider unnecessary. Second, CB encourages people to try unhealthy behaviors and introduce new risk factors into the lifestyle of an individual. Hence, in the absence of CB management, the above process will be repeated continuously, gradually increasing CB and its damage to health behavior intention and ending in maintenance of risk behaviors. As long as CB is not hindered and controlled, the remaining health behaviors will continue to be threatened, and new risk behaviors are likely to form (Petersen et al., 2019). The example mentioned in Section “Model Case” is the typical poor long-term health case caused by CB (Rabiau et al., 2009). CB damages Max’s normal behavior with double measures the next morn. Predictably, this weakens his monitoring of diabetes, eventually worsening his health. Based on this explanation, CB poses a great threat to the long-term health of the patients, especially in the field of chronic diseases including obesity and metabolic diseases.

### Empirical Referents

The empirical referents are defined at the end of the analysis and contain measurements of attributes of the concept. The empirical referents of CB have high practical significance since they can help practitioners and caregivers to identify whether a patient has such beliefs or to evaluate the level of CB.

The earliest CHB scale, which was put forward by Knäuper et al. (2004), has 17 items and is divided into 4 dimensions: eating/sleeping habits, weight regulation, stress, and substance use. In 2012, Kaklamanou et al. (2013) conducted a think-aloud study where 43 participants completed the CHB scale while thinking to identify the difficulties people encountered in completing the scale and suggest necessary and practical measures to improve the reliability and validity of the scale. The authors provided the following suggestions: (1) designing items for particular behaviors; (2) clearly informing participants that their own beliefs were being measured rather than those of others; (3) maintaining consistency in the words used throughout the scale to prevent confusion in subjects; and (4) distinguishing the measurement of CB and behavior.

Since then, many scholars have adapted and tested the CHB scale under different situations. In the field of weight management, Poelman et al. (2013) developed the diet-related CHB (Diet-CHB) scale, which included more specific descriptions of the type and quantity of foods. They reported three factors associated with diet, including exercise, food portions, and front-of-package labeling. Their findings revealed a significant association between Diet-CHB scale and CHB scale, likely indicative of similarities in CB among people in the field of weight management. Furthermore, Diet-CHB scale also obtained the ideal reliability and validity in the verification.

For other health-related behaviors, researchers have developed various scales based on the CHB scale. For instance, Rabiau et al. (2009) developed a glucose testing CB scale with six items. Some adolescents with diabetes with this belief do not adhere to glucose testing and consider that taking other measures (such as eating healthier) can compensate. Therefore, this scale has been adopted to evaluate teenagers, which can help nurses monitor those who are potentially disinclined to conduct self-glucose monitoring. The score of the scale can also guide clinicians or psychologists to better adjust the therapeutic regimen of their patient. Radtke et al. (2011) designed a smoking-specific CHB scale, which has been shown to have high reliability and validity. Their results further suggested that a smoking-specific CHB scale is an effective tool to assess the readiness of adolescents to stop smoking. Additionally, smoking-specific CHB scale highly correlated with CHB scale, since all its items covered various compensatory strategies. In a recent study, Neter and Bagants (2020) developed the CHBs on the breastfeeding (CHB-BF) scale. Their work extends CB research onto breastfeeding health-related behavior studies. Many women find breastfeeding challenging, and CB helps them escape this cognitive conflict by rationalizing their avoiding breastfeeding. Identifying women with potentially negative attitudes toward breastfeeding using CHB-BF can help to correct their misconceptions in the early postpartum period.

### Conceptualization of CB

This study identified that there are various definitions of CB in the context of health behaviors and that these definitions need to be more precise. After analyzing the existing literature, we propose a final, clarified, and refined definition. CB is:

*A belief that the negative effects of participating in unhealthy behaviors or evading healthy behaviors can be offset by the positive effects of another behavior that the subject believes to be beneficial, thus relieving the inner unease of an individual.*

**FIGURE 4:**
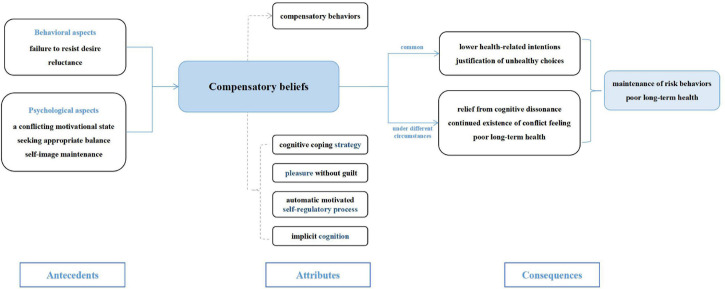
Proposed conceptual model of CB.

Figure 4 shows a conceptual model covering the relationship between attributes, antecedents, and consequences of CB on the theoretical level.

## Discussion

Our study aimed to generate a clarified definition of CB for the management of unhealthy behaviors and to facilitate further interventions for people with such beliefs. CB is characterized by five attributes: cognitive coping strategy, pleasure without guilt, compensatory behaviors, automatic motivated self-regulatory process, and implicit cognition. Clinicians and caregivers can identify patients who have CB by the abovementioned attributes and implement measures to strengthen the management of these patients.

According to our review, it is evident that, in most cases, the activation of CB will have negative effects on the health-related behaviors of the people; hence, developing effective coping strategies that target CB is essential. Although no studies have specifically focused on interventions for negative CB, researchers have offered some recommendations. First, we should promote awareness of their tendency to form such beliefs in individuals with CB (Ernsting et al., 2013) and let them realize these beliefs need to be combined with the execution of behaviors to be effective (Radtke et al., 2011). Second, we can use strategies like cognitive behavioral therapy (CBT) to reconstruct cognitive dissonance (Kronick et al., 2011). In this process, practitioners should help subjects feel responsible for their actions and able to control them (Knäuper et al., 2004). Third, health education targeted compensatory behaviors would be useful for patients to fully realize the detrimental effects of unhealthy behaviors (Forestier et al., 2020). Fourth, steps should be taken to strengthen time management in those with higher CB levels, such as setting a specific compensatory behavior implementation plan and keeping a diary of compensatory behavior completion (Gray et al., 2018).

According to a few other scholars, CB may play a positive role in the cases of individuals with extremely deficient self-control. Amrein et al. found CB is more effective in promoting compensatory behavior than in giving up unhealthy behavior (Amrein et al., 2017). Based on CB, one can first promote the formation of compensatory behavior, then quit harmful behaviors. Similarly, Gray et al. (2018) also observed that, for people who cannot resist temptations at all, promoting CB may help them facilitate healthy behaviors. However, these recommendations of using CB to promote healthy behavior are controversial, as many studies have demonstrated a negative relationship between CB and behavioral motivation (Radtke et al., 2014; Forestier et al., 2020).

This conceptual analysis is of great importance for medical work. Clinicians and caregivers should be aware of the obstruction of CB to the implementation of health behaviors due to its severe negative effects on the medical adherence of patients (Capstick et al., 2019). Our review comprehensively analyzed the concept of CB, which is conducive to a better understanding of the concept and to identify people with CB by the attributes and antecedents we have described. In addition, our review summarized the development history, the existing literature, and the tools for CB evaluation in the past two decades. Taken together, this information can aid researchers interested in CB in learning in which areas there is sufficient empirical evidence of CB and which areas lack in-depth research on health-related CB.

The novelty in our study lies in that we are the first to analyze the important concept of CB with the method of concept analysis. Our review is also a summary of the studies on CB. The literature information table and timeline diagram summarize well the development of CB in recent years. Most importantly, updating the attributes, antecedents, and consequences will bring a new perspective to future research of CB. Our findings make a valuable contribution to better defining and understanding the concept of CB.

Finally, several limitations to this analysis need to be acknowledged. First, empirical studies that explore mechanisms of compensation in health behaviors are lacking. Martin et al. and Petersen et al. were the first to attempt to clarify the mechanism of compensation in weight management (Martin et al., 2019; Petersen et al., 2019). They directly observed the behavioral choices of subjects under the activation of CB in an experimental scenario and their findings corroborate our current knowledge of CB. However, these two studies were focused on diet and exercise behaviors. Therefore, the mechanisms obtained do not apply to the entirety of health management. Second, this review is limited to the field of health behavior management, even though, according to the search results, CB has been reported in other areas as well. For instance, drivers may think: “as long as I slow down, it will be safe to use the cell phone while driving” (Zhou et al., 2016), while environmental managers may think that “it’s OK to cut down trees occasionally as I have been working on projects to improve the ecosystem” (Sörqvist and Langeborg, 2019). Hence, an increasing number of studies have confirmed the existence of CB in the field of health management and other fields. Third, since we only analyzed CB in an English context, a language bias may exist and other language backgrounds should be investigated. In addition, most of the studies we included came from Europe and North America, as we did not find enough CB studies from developing countries. Therefore, studying CB in different social contexts is also necessary because socioeconomic conditions can greatly influence the behaviors of the people.

To explore the mechanisms of CB in greater detail, more influencing factors should be included in future research and practice, and the action path should be discussed or verified based on various theoretical models. Furthermore, researchers need to distinguish between CB and compensatory behaviors because beliefs and behaviors are different dimensions. We recommend focusing on beliefs, because only when the CB is corrected can people sustain healthy behaviors and avoid the maintenance of risk behaviors in the long term. It is necessary to promote positive behavioral change by taking certain interventions that correct false beliefs. In addition, the opposite compensatory effects between behaviors should be taken into account, since individuals may take advantage of their active participation in healthy behaviors to prove that they can engage in unhealthy behaviors (Amrein et al., 2020). Finally, keeping a watchful eye on the potential emotions and cognition in compensatory behavior and paying attention to their distinctions is paramount. This will enable identifying between exercising due to dissatisfaction with body image (Nolan and Eshleman, 2016) from exercising due to eating disorders. Overall, future research on CB should be more detailed and consider CB in different contexts.

## Conclusion

Compensatory belief is one of the principal psychological variables in the health-related behavior management of the patients. The identification, assessment, and regulation of this belief are important for medical work. CB stems from motivational dissonance or confliction. It provides feelings of relief and pleasure but has negative effects on long-term health. The attributes, antecedents, consequences, empirical references, and definitions of CB that were identified in this study can help clinicians and caregivers to develop interventions for CB regulation based on theories, as well as to measure the level of CB in the context of health behaviors management. This concept analysis provides valuable insights that can be used in medical practice, research, education, and management.

## Author Contributions

KZ and QX: study design. KZ: data acquisition and analysis. KZ, QX, HZ, and XX: manuscript preparation. All authors contributed to the article and approved the submitted version.

## Conflict of Interest

The authors declare that the research was conducted in the absence of any commercial or financial relationships that could be construed as a potential conflict of interest.

## Publisher’s Note

All claims expressed in this article are solely those of the authors and do not necessarily represent those of their affiliated organizations, or those of the publisher, the editors and the reviewers. Any product that may be evaluated in this article, or claim that may be made by its manufacturer, is not guaranteed or endorsed by the publisher.
